# Characterizing mortality in patients with AQP4‐Ab+ neuromyelitis optica spectrum disorder

**DOI:** 10.1002/acn3.52092

**Published:** 2024-06-17

**Authors:** Anna Francis, Emily Gibbons, Jeffrey Yu, Karissa Johnston, Hannah Rochon, Lauren Powell, Maria Isabel Leite, Saif Huda, Adrian Kielhorn, Jacqueline Palace

**Affiliations:** ^1^ Oxford University Hospital Oxford UK; ^2^ The Walton Centre NHS Foundation Trust Liverpool UK; ^3^ Alexion, AstraZeneca Rare Disease Boston Massachusetts USA; ^4^ Broadstreet Health Economics & Outcomes Research Vancouver British Columbia Canada; ^5^ Department of Clinical Neurosciences Oxford University Hospitals Oxford UK; ^6^ Guy's and St Thomas's NHS Foundation Trust Liverpool UK

## Abstract

Neuromyelitis optica spectrum disorder is an autoimmune disease, causing severe disability due to relapses, but recent mortality data are limited. Among 396 patients seropositive for anti‐aquaporin‐4 antibody from 2014 to 2020 in the United Kingdom, 39 deaths occurred: 19 (48.7%) were unrelated to disease; 14 (35.9%) were severe disability‐ or relapse‐related; and 4 (10.3%) were attributed to malignancy/infection. Mean annual mortality was 1.92% versus 0.63% in the matched population. The standardized mortality ratio was 3.04 (95% confidence interval 1.67–5.30) with 1.29% excess mortality per year in patients. Median Expanded Disability Status Scale before death was 7.0. Results highlight the importance of preventing relapses that drive disability.

## Introduction

Neuromyelitis optica spectrum disorder (NMOSD) is a rare, chronic autoimmune disease of the central nervous system, characterized by unpredictable relapses that may lead to blindness, paralysis, cognitive impairment, and death.^1^, ^2^, ^3^ Most patients have serum immunoglobulin‐G (IgG) to the aquaporin‐4 protein (AQP4‐Ab+).^4^ Relapses and consequent disability result in high costs related to pharmacy/medications, ambulatory/outpatient treatments, and hospitalizations.^5^, ^6^, ^7^, ^8^ Quality of life and psychological health are also negatively impacted by NMOSD‐related disability.^9^

NMOSD was associated with high mortality before the improvement in diagnosis and widespread use of immunosuppressant therapies.^2^ Since then, only a few NMOSD mortality studies have been conducted, but they have shown evidence of declining mortality rates.^2^ Contemporary mortality rates range from ~2%–7% per year, but these studies did not adjust for background mortality.^10^, ^11^, ^12^, ^13^ Although the natural disease course of NMOSD has been characterized,^1^, ^14^ the relationship between disability and mortality in patients with NMOSD is not fully understood.

The objective of this study was to examine mortality and excess mortality in patients with NMOSD compared with the United Kingdom's (UK's) general population.^15^ Cause of death was characterized, and disability before death was assessed in relation to disease duration.

## Methods

Prospectively entered data from two databases were retrospectively analyzed for this study: the UK National NMOSD data set maintained by Oxford University Hospital (Oxford, UK) and the Walton Centre NHS Foundation Trust (Liverpool, UK) over a 7‐year period (2014–2020). The NHS data source was also reviewed for any patients not seen recently. AQP4‐Ab+ status was determined using live cell‐based assay.^4^ The Oxfordshire Research Ethics Committee (Ref: 21/SC/0353) and NRES Committee London—Hampstead (Ref: 15/LO/1433) reviewed the study. No patient identifiable information was used.

### Main outcomes and measures

#### Mortality

Annual mortality rate was calculated as average yearly mortality observed in the pooled Oxford and Liverpool cohort (i.e., sum of 7 annual mortalities divided by 7 study years). Mortality was characterized by a standardized mortality ratio (SMR) and excess mortality compared with the age‐, sex‐, and race‐matched population (based on 2019 UK data among those aged 18–85 years).^16^ Within each self‐identified race category (i.e., White, Black, Asian, Other), equally distributed ethnic groups were assumed. SMR was calculated by dividing number of deaths in patients by expected number of deaths in the age‐, sex‐, and race‐matched cohort. SMR > 1.0 indicates excess death. Excess mortality was calculated by subtracting expected mortality from observed mortality in NMOSD, where excess mortality was considered > 0%.

#### Cause of death

Cause of death was determined from patients' death certificates or from reports by their treating neurologist, general practitioner, or hospital record. Death from comorbidities not associated with NMOSD was defined as any cause of death without well‐documented NMOSD association. Death was attributed to severe disability if patients had a last‐recorded Expanded Disability Status Scale (EDSS)^17^ score > 6.5 (impaired mobility) and died of disease that would not have occurred or that would have been reasonably easily treated in patients with normal neurological function. Death due to relapse was defined as death arising during and as a result of acute relapse, typically with brain and/or brainstem involvement. Death related to co‐existing autoimmune disease was defined as death due to autoimmune disease reported to be associated with AQP4‐Ab+ NMOSD (e.g., systemic lupus erythematosus, myasthenia gravis, and Sjogren's disease). Immunosuppression‐related death was defined as death from infection that would not ordinarily cause severe disease in a person with an intact immune system or death from cancer that is rare or would not be expected in patients with intact immune systems.

#### Disability

Disability was characterized by a patient's last available EDSS score before their final illness. Scores were based on clinical assessments and rated from 0 (normal neurological exam with no disability) to 10 (death due to NMOSD).^17^

### Statistical analysis

Data were stored in a secure Microsoft Access database and analyzed using IBM SPSS Statistics version 28.0. Linear regression analysis was conducted to assess the relationship between EDSS prior to death with disease duration among patients with NMOSD‐related mortality.

## Results

The overall cohort included 396 patients with AQP4‐Ab+ NMOSD (Table 1). Median age at last year of data collection was 54 years (range: 10–90), and 339 (85.6%) patients were female. Self‐reported race was White in 215 (54.3%), Black in 92 (23.2%), Asian in 62 (15.7%), “other” in 5 (1.3%), and unknown in 22 (5.5%).

**Table 1:** Patient demographics and characteristics.

Characteristic	AQP4‐Ab+ NMOSD Cohort	
	Overall (*n* = 396)	Deaths (*n* = 39)
Age, years		
Median	54	66
Min–max	10–90	16–88
Sex, *n* (%)		
Male	57 (14.4)	6 (15.4)
Female	339 (85.6)	33 (84.6)
Race, *n* (%)		
White	215 (54.3)	29 (75.4)
Black	92 (23.2)	5 (12.8)
Asian	62 (15.7)	3 (7.7)
Other	5 (1.3)	0
Unknown	22 (5.5)	2 (5.1)
Disease duration, years		
Mean ± SD	N/A	10.1 ± 7.1
Median	N/A	10.0
Min–max	N/A	0.7, 31.9
Last EDSS score		
Mean ± SD	N/A	6.8 ± 1.7
Median	N/A	7.0
Min–max	N/A	3.0–9.0

AQP4‐Ab+, seropositive for immunoglobulin‐G (IgG) to the aquaporin‐4 protein; EDSS, Expanded Disability Status Scale; N/A, not available; NMOSD, neuromyelitis optica spectrum disorder; SD, standard deviation.

During the 7‐year study period, 39 (9.8%) patients died (Table 1). Median age at death was 66.0 years (range: 16.0–88.0), and 33 patients (84.6%) were female. Twenty‐nine patients (75.4%) were White, 5 (12.8%) were Black, 3 (7.7%) were Asian, and 2 (5.1) were unknown, indicating White patients were overrepresented in the deceased (75.4%) compared with the total cohort (54.3%). Median disease duration was 10.1 years (range: 0.7–31.9), and EDSS score before death was 7.0 (range: 3.0–9.0). At time of death, 34 (87.2%) patients were on immunosuppressive medications (methotrexate, mycophenolate, azathioprine, cyclophosphamide, rituximab, and/or prednisolone), 4 were not, and 1 patient's treatment could not be confirmed.

**Figure 1:**
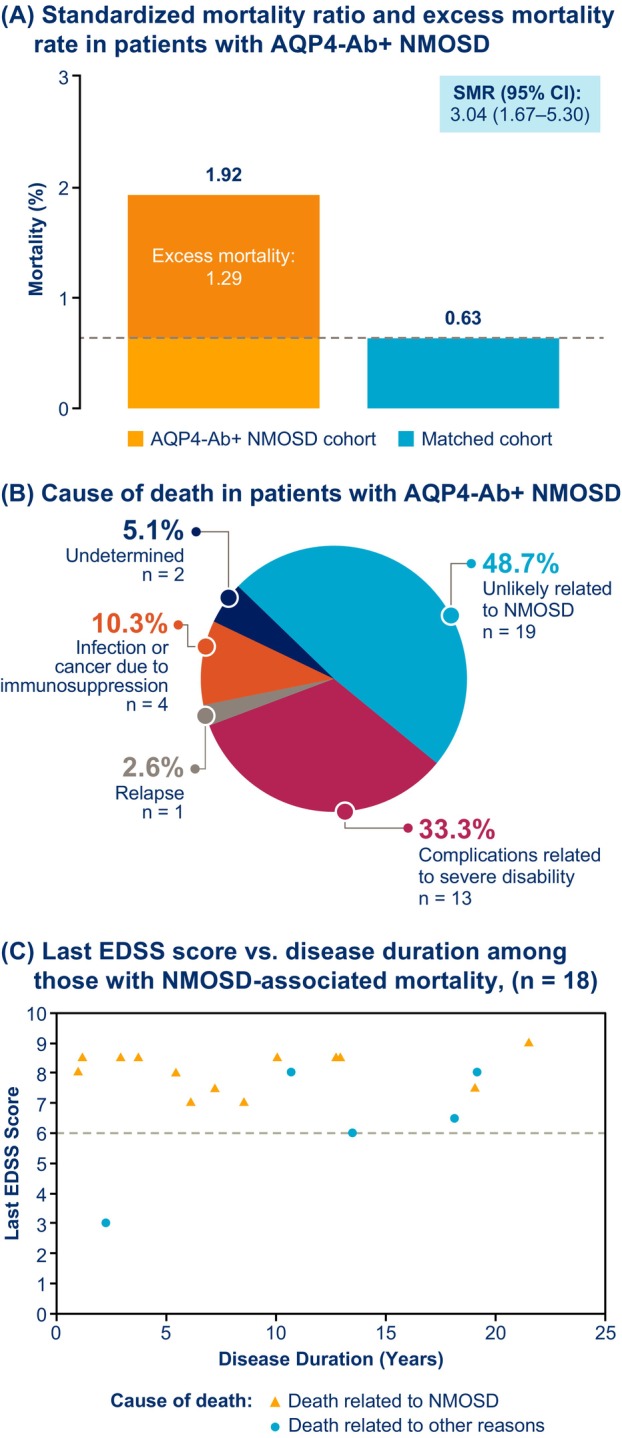
(A) Standardized mortality ratio and excess mortality rate, (B) cause of death, and (C) last EDSS score before death versus disease duration. Death due to relapse is defined as death arising during and as a result of acute relapse. EDSS scores are taken from the last available clinical assessment or report prior to death; *p* = 0.56, derived from a simple linear regression model (*n* = 18). AQP4‐Ab+, seropositive for immunoglobulin‐G (IgG) to the aquaporin‐4 protein; CI; confidence interval; EDSS, Expanded Disability Status Scale; NMOSD, neuromyelitis optica spectrum disorder; SMR, standardized mortality ratio.

Mean annual death rate over the 7‐year period was 1.92% (Fig. 1A). The average weighted mortality rate of the age‐, sex‐, and race‐matched general population was 0.63%, resulting in an SMR of 3.04 (95% confidence interval 1.67–5.30) and excess mortality rate of 1.29% annually.

Death was unrelated to NMOSD in 19 (48.7%) cases (Fig. 1B). Thirteen (33.3%) deaths were attributed to severe disability, largely comprising cases of pneumonia in bed‐bound patients. Death was attributed to relapse in 1 (2.6%) patient and undetermined for 2 (5.1%) patients. No patient died as a result of an associated autoimmune disease.

Four (10.3%) patients died of infection or cancer possibly related to immunosuppression. A 56‐year‐old patient on mycophenolate mofetil and prednisolone died of sepsis secondary to community‐acquired pneumonia (EDSS of 6.0); a 32‐year‐old patient on rituximab and prednisolone died of sepsis (EDSS of 3.0); an 87‐year‐old patient receiving methotrexate and prednisolone died of pneumonia (EDSS of 8.5); and a 51‐year‐old patient on azathioprine and prednisolone died of metastatic bladder cancer (EDSS of 6.5).

Most deceased patients (31/39; 79.5%) had severe disability before death (EDSS ≥ 6). Among 18 patients with NMOSD‐related mortality, EDSS before death was not significantly associated with disease duration (*p* = 0.56; disease duration: mean [SD] = 9.8 [6.6] years, median [range] = 9.3 [1–21.5] years; Fig. 1C).

## Discussion

In this observational UK cohort study, annual mortality rate among patients with AQP4‐Ab+ NMOSD was 1.92%, with an SMR of 3.04 and excess mortality of 1.29% annually when compared with the general population. More deaths were attributed to NMOSD‐related disability or relapse (36%) than immunosuppression (10%). Around 80% of deceased patients had an EDSS ≥ 6.0 before final illness and those with NMOSD‐related mortality had a higher median EDSS of 9.3, independent of disease duration.

The 1.92% annual mortality rate was at the lower end of rates previously reported (2% to 7%^10^, ^11^, ^12^, ^13^), potentially due to differences in cohort demographics including race, age at onset, lower use of immunosuppression, patients treated by comprehensive experts and multidisciplinary care teams commissioned through a national highly specialized service and/or variable proportions of seropositive cases in other studies.^10^, ^11^, ^12^ It is possible that some undiagnosed cases or patients not referred to our service died during the onset attack, and this would mean the NMOSD‐related mortality is underestimated. Still, the 3.04 SMR indicates patient mortality was triple the background level when adjusted for age, sex, and race. Most of the 39 deceased patients had severe disability, and over one‐third of deaths were attributable to disability or relapse, consistent with studies showing high mortality directly attributable to the disease.^10^, ^12^, ^18^ As disability in NMOSD is largely relapse‐related, this highlights the need for early diagnosis and better relapse prevention. The substantial number of deaths unrelated to NMOSD also suggests future work should investigate comorbidities related to NMOSD mortality.

Risk of relapse and subsequent disability (and death) needs to be balanced against the inherent risks of long‐term immunosuppression. We identified 4 deaths possibly attributable to immunosuppression; however, leaving NMOSD untreated leaves patients at high risk of relapse and subsequent severe disability.^5^ Physicians may face challenges in carefully weighing the concerns of developing infection or new comorbidities with continuing effective treatment and decreasing risk of relapse. A higher rate of death due to infection has been observed in patients without appropriate immunosuppression versus those on effective therapy, and the current study also found high incidence of death due to infection in those with high EDSS scores.^10^ Together, this may suggest that disability accumulated as a result of relapses conveys a greater risk of fatal infection than immunosuppression. While none of the deceased patients in this study received novel treatments at the time of death, it is currently unknown how newly available therapies with high efficacy on relapse reduction (e.g., ravulizumab or eculizumab) may influence mortality. Regular surveillance for high‐risk malignancies, appropriate vaccination schedules, and monitoring for infection are important, especially considering immunosenescence in the elderly.

There was a greater proportion of White patients among those who died compared with the total AQP4‐Ab+ NMOSD cohort, consistent with studies showing greater levels of motor disability in White cohorts (which may be attributable to older age at disease onset) as well as higher mortality in White patients.^1^ The healthy immigrant effect may also play a role, where the White UK‐born population is more likely to have health problems than migrants in the UK.^19^ Furthermore, it should be acknowledged that the UK cohorts evaluated in this study may not be fully representative of the demographic and clinical characteristics of other populations of patients with NMOSD, which may influence the interpretation of these findings. It should be noted that while racial differences have been observed for age at onset and number of severe attacks in a worldwide study of patients with NMOSD, severe motor disability was most dependent on early and effective immunosuppressive treatment.^20^

The study included a limited number of patients, and therefore deaths, to examine. Death related to disability or immunosuppression was inferred from clinical history but cannot be confirmed pathologically. Timing of death in relation to the time at which disability was recorded varied between patients, leading to a possible underestimation of EDSS scores in some cases. However, other than the patient who died during an acute relapse, there were no intervening relapses between final recorded EDSS score and death.

While NMOSD is known to severely affect morbidity, these data show that it still is associated with increased mortality, with one‐third of deaths related to disability. These findings suggest that patients with AQP4‐Ab+ NMOSD could benefit from more effective and safer relapse‐preventing therapies throughout the disease course in addition to preventing and treating contributing factors to severe infections.

## Author Contributions

Conception and design of the study: AF, JP, AK, JY, and SH. Acquisition and analysis of data: AF, EG, JY, KJ, HR, LP, MIL, SH, AK, and JP. Drafting a significant portion of the manuscript or figures (i.e., a substantial contribution beyond copy editing and approval of the final draft, which is expected of all authors): AF, JY, SH, KJ, HR, LP, AK, and JP.

## Conflict of Interest

Anna Francis has received travel grants from Merck and Alexion. Adrian Kielhorn is an employee and shareholder of Alexion, AstraZeneca Rare Disease. Emily Gibbons and Saif Huda have nothing to disclose. Jeffrey Yu is an employee and shareholder of Alexion, AstraZeneca Rare Disease. Karissa Johnston, Hannah Rochon, and Lauren Powell are the employees of Broadstreet HEOR, which received funding from Alexion, AstraZeneca Rare Disease to conduct this work. Maria Isabel Da Silva Leite is funded by the NHS (Myasthenia and Related Disorders Service and National Specialized Commissioning Group for Neuromyelitis Optica, UK) and by the University of Oxford, UK. She has been awarded research grants from the UK Association for patients with Myasthenia (Myaware), Muscular Dystrophy Campaign (MDUK), and the University of Oxford. She has received speaker honoraria and travel grants from UCB Pharma and Horizon Therapeutics, and consultancy fees from UCB Pharma. She serves on scientific or educational advisory boards for UCB Pharma, Argenx, and Horizon Therapeutics. Jacqueline Palace has received support for scientific meetings and honoraria for advisory work from Merck Serono, Novartis, Chugai, Alexion, Roche, Medimmune, Argenx, Vitaccess, UCB, Mitsubishi, Amplo, and Janssen. Grants from Alexion, Argenx, Clene, Roche, Medimmune, Amplo biotechnology. Patent ref P37347WO and license agreement Numares multimarker MS diagnostics Shares in AstraZeneca. Her group has been awarded an ECTRIMS fellowship and a Sumaira Foundation grant to start later this year. A Charcot fellow worked in Oxford 2019–2021. She acknowledges partial funding to the trust by highly specialised services NHS England. She is on the medical advisory boards of the Sumaira Foundation and MOG project charities, is a member of the Guthy‐Jackson Foundation Charity and is on the Board of the European Charcot Foundation and the steering committee of MAGNIMS and the UK NHSE IVIG Committee and chairman of the NHSE neuroimmunology patient pathway and ECTRIMS Council member on the educational committee since June 2023. On the ABN advisory groups for MS and neuroinflammation and neuromuscular diseases.

## Funding Information

Funding was provided by Alexion, AstraZeneca Rare Disease, Boston, MA, USA.

## Data Availability

Alexion will consider requests for disclosure of registry participant‐level data provided that participant privacy is assured through methods like data de‐identification, pseudonymization, or anonymization (as required by applicable law), and if such disclosure was included in the relevant study informed consent form or similar documentation. Qualified academic investigators may request participant‐level clinical data and supporting documents (statistical analysis plan and protocol) pertaining to Alexion‐sponsored studies. Further details regarding data availability and instructions for requesting information are available in the Alexion Clinical Trials Disclosure and Transparency Policy https://www.alexionclinicaltrialtransparency.com/data‐requests/. Link to Data Request Form (https://alexion.com/contact‐alexion/medical‐information).
